# Tobacco use, smoking identities and pathways into and out of smoking among young adults: a meta-ethnography

**DOI:** 10.1186/s13011-022-00451-9

**Published:** 2022-03-28

**Authors:** Ria Poole, Hannah Carver, Despina Anagnostou, Adrian Edwards, Graham Moore, Pamela Smith, Fiona Wood, Kate Brain

**Affiliations:** 1 European Centre for Environment and Human Health, University of Exeter Medical School, Royal Cornwall Hospital, Knowledge Spa, Cornwall TR1 3DH Truro, UK; 2 Salvation Army Centre for Addiction Services and Research, Faculty of Social Sciences, University of Stirling, FK9 4LA Stirling, UK; 3 Division of Human Health Sciences, Graduate School of Medicine, Kyoto University, 53 Shogoin Kawahara-cho, Sakyo Ward, 606-8507 Kyoto, Japan; 4 Division of Population Medicine, School of Medicine, Cardiff University, Neuadd Meirionydd, Heath Park, CF14 4YS Cardiff, UK; 5 Centre for the Development and Evaluation of Complex Interventions for Public Health Improvement (DECIPHer), School of Social Sciences, Cardiff University, 1-3 Museum Place, CF10 3BD Cardiff, UK

**Keywords:** Meta-ethnography, Systematic review, Young adults, Tobacco use, Smoking prevention, Smoking cessation, Smoking identities

## Abstract

**Background:**

This meta-ethnography investigates how young adults describe their tobacco use, smoking identities and pathways into and out of regular smoking, to inform future smoking prevention and harm reduction interventions.

**Methods:**

Eight databases were systematically searched using keywords and indexed terms. Studies were included if they presented qualitative data from young adults aged 16–25 reporting smoking histories and/or smoking identities from countries culturally similar to the UK. A systematic and rigorous meta-ethnographic approach was employed, consistent with Noblit and Hare’s methodology.

**Results:**

Thirty papers were included. Reasons stated for taking up smoking and becoming a smoker included alleviating stress, transforming one’s identity, and coping with the transition to further education, employment or leaving home. Many used smoking to aid acceptance within new peer groups, particularly when alcohol was present. Smoking was also perceived as an act of resistance and a coping mechanism for those with marginalised identities. Barriers to quitting smoking included young adults’ minimisation or denial of the health risks of smoking and not identifying with “being a smoker”.

**Conclusions:**

This meta-ethnography may provide a blueprint to inform the development of health and wellbeing interventions designed specifically for young adults. Smoking cessation interventions should be co-designed with young adults based on their perceived needs, resonant with their desire to quit in the future at key milestones. Harm reduction interventions should address the social aspect of addiction, without reinforcing stigma, particularly for those with marginalised identities.

**Supplementary Information:**

The online version contains supplementary material available at 10.1186/s13011-022-00451-9.

## Introduction

Recent studies into smoking have begun to identify a complex typology of smoking identities and varied patterns of tobacco use during the transition to adulthood including large numbers of “casual” smokers and “social” smokers - populations not well served by current cessation approaches [1–6]. As the minimum legal age required to purchase tobacco in the UK rose from 16 to 18 in 2007, and is likely to be raised again to 21 in England [7], legislation has been a key driver to reduce smoking uptake in the UK as it has been in the US [8]. In 2019 the US Congress raised the federal minimum legal sales age for tobacco products from 18 to 21 years [9]. In most European countries, the minimum age to purchase tobacco is 18, with the exception of Austria and Belgium where the minimum age is 16; however, in 21 EU Member States, there is no minimum age for being permitted to consume tobacco [10]. In the UK, a Parliamentary report published in 2021 highlighted the importance of increased funding, legislation, and behaviour change policy and interventions for the UK Government to deliver a ‘Smokefree 2030’ [11]. Elsewhere, Australia has tended to be ahead of the UK regarding tobacco policy, as it has introduced smoke free legislation, plain packaging and banned smoking in cars carrying children among other initiatives before the UK [12]. New Zealand has recently announced some of the most radical policy moves, such as prohibiting the sale, delivery and supply of smoked tobacco products to individuals born after a certain date, with an intention to phase out tobacco entirely by 2025 [13].

In the UK, recent data showed that 32% of current and ex-smokers aged 16–24 started when they were 16 or 17 years old [7], during the turbulent and transitionary phase of the lifecourse from youth into young adulthood which is characterised by the disruption of old habits and behaviours and the establishment of new ones [14, 15]. In order to inform appropriate targeted interventions (aged 16–25), it is essential to understand how young adults construct and negotiate their identities in the context of smoking, and how and why they transition into and out of smoking regularly.

The meta-ethnography approach brings together standalone qualitative studies to provide a new interpretation of the evidence, and is useful in informing service delivery and designing suitable interventions for this target population [16]. The classic meta-ethnography was designed by Noblit and Hare as a methodology to synthesise primary qualitative studies in an interpretative and theory-generating way [16]. Their seven phase approach requires a systematic comparison of data across studies to develop new overarching concepts, theories and/or models for the phenomena of interest [16]. The meta-ethnography differs from other qualitative synthesis approaches because, rather than purely reporting on or aggregating identified themes from a qualitative synthesis, the meta-ethnography’s systematic process preserves the context and meanings of the primary studies, whilst using a unique translation synthesis method in order to transcend the findings of individual study accounts and create higher order themes, leading to a new interpretation or conceptual understanding which can better inform intervention development [17]. A meta-ethnographic synthesis approach is suitable when there is specific interest in a conceptual or theoretical understanding of a particular phenomenon.

Researchers have noted, however, that Noblit and Hare’s methodology may be interpreted in different ways and as a result meta-ethnographies vary widely in practice, reporting and quality [18]. To address this concern, new reporting guidelines for reporting meta-ethnography may facilitate high quality, transparent reporting for the methods, analysis and findings [18].

A meta-ethnography conducted by Tombor et al. (2015) investigated the potential role of smoker identity in young adults’ smoking behaviour and how identity change could influence smoking and cessation attempts [19]. The authors identified a complex array of smoking identities and the role of identities, motives and contextual factors in influencing quit attempts [19]; however, experiences of tobacco use, patterns and trajectories were not looked at specifically. Therefore, if we are to tailor interventions to this target population, it is crucial to fully understand how young adults’ identities develop and shift as they journey into adulthood in relation to their smoking identities and motivations for using tobacco. Beyond the individual studies, there is a story to be told that makes sense of the evidence as a whole, whilst striving to preserve and honour any contextual or demographic differences which were identified as relevant. Our meta-ethnography investigated how young adults describe their tobacco use, smoking identities and pathways into and out of regular smoking, for the purpose of generating new theoretical insights into the timing, nature and influences upon young adults’ smoking trajectories. As such, we addressed the following questions:

1. How do young adults describe their tobacco use?
2. How do smoking identities and lifecourse transitions into adulthood influence pathways into and out of regular smoking?

Findings will identify smoking cessation and harm reduction strategies to inform future intervention development. As a Cochrane systematic review has already found limited evidence that either behavioural support or smoking cessation medication increases the proportion of young people that stop smoking in the long-term [20], our study focuses on harm reduction as a strategy most appropriately suited to this target population. National Institute for Health and Care (NICE, UK) guidance on preventing smoking uptake, promoting quitting and treating dependence defines harm reduction as measures to reduce the illnesses and deaths caused by smoking tobacco among people who smoke and those around them [21].

This meta-ethnography is the first to synthesise the international literature to understand young adults’ smoking trajectories to understand the potential key components required in future interventions for this group and to inform the wider evidence base.

## Methods

A systematic and rigorous meta-ethnographic approach was employed, consistent with Noblit and Hare’s methodology [16] and with reference to the eMERGe Guidelines for reporting meta-ethnographies [18]. The seven steps for conducting a meta-ethography as described by Noblit and Hare are indicated within each sub-heading below [16].

### Search strategy

ASSIA (Applied Social Sciences Index and Abstracts), CINAHL (Cumulative Index to Nursing and Allied Health Literature), Science Citation Index, Social Science Citation Index, Embase, Medline, Medline-in-Process and PsycINFO databases were systematically searched using a mix of keywords and indexed terms from 1998 (when youth smoking began to decline in the UK) [22, 23] up to September 2020. A search strategy was designed in Ovid Medline and has been translated for other databases (see Additional File 2 for search terms). For included papers citations were identified and reference lists reviewed. This process is encompassed within Phase 1 of Noblit and Hare’s methodology (‘Getting started’) [16].

### Inclusion and exclusion criteria

Phase 2 of Noblit and Hare’s methodology is ‘Deciding what is relevant’ [16]. Studies were included if they were: primary qualitative studies; published in the English language since 1998 [22, 23]; reporting data predominantly from young adults aged 16–25; reporting smoking histories and/or smoking identities; from countries culturally similar to the UK (USA, Canada, Australia, New Zealand, Western Europe) indicated by scoring highly on dimensions of secular-rational values (compared to traditional values) and self-expression values (compared to survival values) within the Inglehart-Weizel World Cultural map to depict analysis of the World Values Survey data [24], and also because of expected (and therefore informative) heterogeneity of studies in these countries which are similar in terms of tobacco control policy (such as age of sales and introducing smoke-free legislation) and smoking rates [25]. The lead author (RP) conducted title and abstract screening to select potential studies for inclusion and the relevance of included selected studies was assessed using the study inclusion criteria. At the full text screening stage, if a study was considered to be lacking sufficient data to extract first and second order concepts (participant quotes and author’s interpretations, respectively), then it was reviewed by a second researcher (HC) to consider its suitability for inclusion. All studies selected for possible inclusion were read in full by RP and all inclusion decisions were confirmed by KB. This is consistent with Noblit and Hare’s Phase 3 (‘Reading the studies’) [16].

### Quality assessment

The rigour and credibility of included studies were independently evaluated by members of the research team (RP, DA, KB, PS) using the CASP [26] (Critical Appraisal Skills Programme) checklist for qualitative studies. The CASP Qualitative Critical Appraisal Tool is the most commonly used and robust qualitative critical appraisal tool used for qualitative evidence synthesis and is recommended by the Cochrane Qualitative and Implementation Methods Group [27–29]. The Critical Appraisal Skills Checklist (CASP) provides detailed instructions and decision rules on how to interpret the suggested criteria [26]. This checklist is designed to help the reviewer assess the rigour, credibility and relevance of each study and has been successfully used in other published meta-ethnographies [30–33]. We are aware that other tools exist [34, 35], some of which focus on more conceptually rich index accounts; however, the different ways of ordering study accounts has yet to be formally empirically compared and there is no guidance for reviewers [36, 37]. Furthermore, in a systematic mapping of existing tools to appraise methodological strengths and limitations of qualitative research it was found that many other recent critical appraisal tools for qualitative research are published adaptations of existing checklists developed without hypotheses or empirical evidence to investigate the relationships between components of qualitative study design, conduct or the trustworthiness of findings [35].

Minor disagreements regarding the quality rating were resolved through discussion. Eighteen of the included papers were considered of high quality, nine were considered medium quality and three of medium to low quality. All papers were included within the synthesis due to their conceptually rich first and second order data.

### Data extraction (selection and coding)

Consistent with Noblit and Hare’s Phase 3 (‘Reading the studies’) and with their Phase 4 (‘Determining the relationships between the studies’), we performed data extraction and coding [16]. At the beginning of the data extraction process two researchers (RP, HC) independently read and extracted first and second order data from the first two studies in parallel, checking for any uncertainties or discrepancies in the data. They extracted first and second order data from the remaining studies independently. The first and second order concepts of two papers were independently analysed for comparison and discussion and to facilitate the consistent application of the data extraction method. Key characteristics of study design, participant characteristics, results (including themes and quotes) and authors’ interpretations were extracted and coded in NVivo 11 [38].

### Translating interpretations and synthesis

Data synthesis was conducted across the seven phases, working backwards and forwards across the phases and overlapping phases as necessary [16]. Translation of studies (Phase 5) was achieved through reciprocal analysis (for similar cases) rather than refutational analysis (for disconfirming cases) because interpretations across studies were similar throughout first, second and third order interpretations [16]. The studies were compared and contrasted iteratively and the lead author discussed emerging interpretations with the study team. The lead author conducted a thematic analysis for the first order and second order data (quotes and authors’ interpretations, respectively), logging reflections throughout, and at least one other researcher (KB, HC) reviewed a proportion of the transcripts to enable discussion of concepts and any contradictory findings. Studies were grouped according to participant characteristics (such as gender and cultural identification), according to the available data. Across Phases 6 (‘Synthesising the translations’) and 7 (‘Findings’), findings were tabulated for review and discussion within the study team, where alternative interpretations were also considered, and the data were mapped to second order interpretations (i.e. synthesising the data to create the next level (third order) of themes) [16]. To preserve the context and meaning of the relationships between concepts within and across studies, the key descriptors used within the first and second order interpretations were carried over to the third order interpretations (the interpretations of the synthesis team expressed as themes and key concepts), where appropriate to do so.

For the third order interpretations, two researchers (RP, KB) independently considered the first and second order interpretations to derive the new, overarching concepts and conceptual categories that comprise the “line of argument” synthesis. To encompass the behavioural determinants, environmental factors and health considerations of young adults who smoke, we chose to take an ecological approach to the findings within the line of argument synthesis and used the PRECEDE theoretical framework as a lens to convey the pathways of the problem at multiple ecological levels (interpersonal, organisational and societal) [39, 40]. The PRECEDE model has been used as the basis for health intervention planning across a multitude of studies, and recognises the lived experiences of stakeholders as fundamental to defining the problem and thus to informing potential solutions [39, 41–44].

## Results

**Fig. 1:**
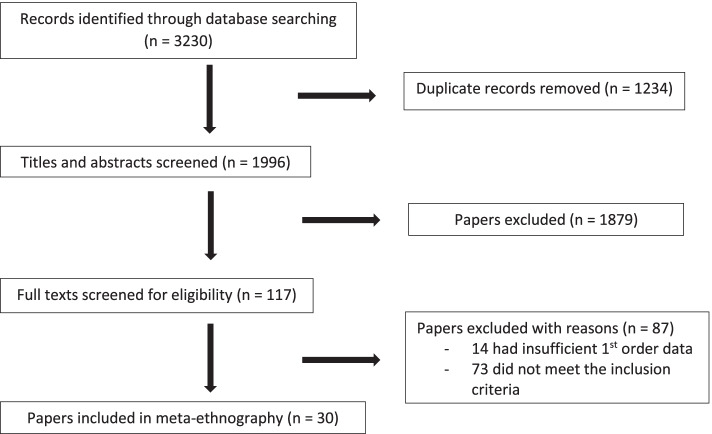
PRISMA flowchart of study selection. PRISMA flowchart displays study selection along with reasons for paper exclusion

The study selection process is shown in Fig. 1. Thirty papers were identified which fulfilled our inclusion criteria.

Characteristics of included studies are detailed in Table 1. Included studies took place in nine countries (Finland, Scotland, USA, Ireland, Australia, England, Canada, New Zealand and Norway), and were published between 2000 and 2020. The self-reported smoking statuses of participants were noted within each study and these included current smokers, former smokers, ever-smokers and never smokers. Sample sizes ranged from 13 to 99 participants, and data collection methods were primarily focus groups and interviews. A variety of qualitative analysis methods were employed, to include thematic analysis, content analysis, framework analysis, narrative analysis and grounded theory among others. Papers by Scheffels (2009) and Scheffels and Schou (2007) were derived from the same primary study, using the same qualitative interview data collected in 2002 [45, 46]. Similarly, papers by Amos et al. (2006) and Wiltshire et al. (2005) were derived from the same primary study, drawing on the same set of qualitative interview data and also responses to a brief structured questionnaire [47, 48].

**Table 1:** Characteristics of included studies

Paper No.	Authors (date of publication), country, reference	Research aims	Population, age, gender, cultural identification, smoking status	Qualitative data collection methods	Qualitative analysis
1	Aho et al. (2019), Finland [49]	To assess how practical nursing students perceive themselves as smokers and future healthcare workers	*N* = 29 / Ages 16–25 / Gender identification: not reported / Racial and cultural identification: not reported / current smokers	6 focus groups	Critical discourse analysis
2	Amos et al. (2006), Scotland, UK [47]	To explore Scottish 16–19 year olds’ understanding of their smoking and attitudes towards quitting and cessation support	*N* = 99 / Ages 16–19 / Female: 52 / Male: 47 / Racial and cultural identification: not reported / current smokers	Mostly paired interviews, 4 three-person group interviews and 1 one-to-one interview	A modified grounded theory approach
3	Antin et al. (2017), USA [50]	To investigate the lived experience of smoking stigma for black women who smoke and understand the potential unintended consequences of tobacco denormalisation policies for low-income black women	*N* = 15 / Ages 18–25 / Female: 14 / Not identifying with any particular gender: 1 / Black American: 15 / current smokers	10 one-to-one interviews and 1 group interview	Thematic analysis
4	Antin et al. (2018), USA [51]	To examine the meanings of tobacco in the lives of sexual and gender minority youth in San Francisco Bay Area	*N* = 58 / Ages 19–25 / Sexual and gender minority (SGM): 58 / “Ethnic minority”: 78% / current smokers	58 one-to-one interviews	Narrative analysis
5	Berg et al. (2010), USA [52]	To examine how college students define the term ‘smoker’ and how this definition impacts their behaviour and attitudes	*N* = 73 / Ages 18–25 / Female: 41 / Male: 32 / Non-Hispanic white: 65 / Other racial identity: 8 / current smokers	12 focus groups	Focus group analysis
6	Breslin et al. (2018), Ireland [53]	To explore and understand the factors associated with young people’s use of roll-your-own tobacco	*N* = 62 / Ages 16–22 / Female: 29 / Male: 33 / Racial and cultural identification: not reported / current smokers	22 one-to-one interviews and 8 focus groups	Categoric and thematic data analysis
7	Brown et al. (2011), USA [54]	To examine the motivations behind occasional smoking within college students who often do not define themselves as smokers	*N* = 53 / Ages 18–25 / Female: 30 / Male or not specified: 22 / Caucasian: 27 / Other racial identity: 8 / occasional smokers	8 focus groups	Thematic analysis
8	Cheney et al. (2017), USA [55]	To understand how the Greek fraternity and sorority university social networks influence smoking attitudes, beliefs and behaviours among their members	*N* = 33 / Ages 18–25 / Sorority members: 16 / Fraternity members: 17 / White: 32 / White Hispanic: 1 / current smokers	33 one-to-one interviews	Thematic analysis
9	Delaney et al. (2018), Scotland, UK [56]	To explore young adults’ perceptions and experiences of smoking and their smoking trajectories in the context of their social and occupational histories and transitions in Scotland	*N* = 15 / Ages 20–24 / Female: 6 / Male: 9 / Racial and cultural identification: not reported / ever-smokers	15 one-to-one interviews	Thematic analysis
10	Dono et al. (2020), Australia [57]	To explore how social relationships and normative group behaviours can be barriers to transitioning from a smoker to non-smoker identity	*N* = 30 / Ages 18–25 / Female: 13 / Male: 17 / Racial and cultural identification: not reported / daily smokers, occasional smokers and former smokers	6 focus groups	Thematic analysis
11	Foraker et al. (2005), USA [58]	To assess the beliefs and attitudes regarding tobacco use interventions among young adult Latinos	*N* = 19 / Ages 18–24 / Female: 12 / Male: 7 / Latino: 19 / current smokers, former smokers and never smokers	Individual and group interviews	Content analysis
12	Fry et al. (2008), England, UK [6]	To explore the reasons why smokers and non-smokers believe young people smoke, focusing on how and why they believe that they start, continue, and problems perceived with stopping smoking	*N* = 87 / Ages 16–24 / Female: 46 / Male: 39 / Racial and cultural identification: not reported / current smokers and non-smokers	22 focus groups	Framework analysis
13	Gilbert (2007), Australia [59]	To explore what cigarette smoking means to young women in their adolescent years and to see if the decision to begin smoking is related to identify formation	*N* = 20 / Ages 18–24 / Female: 20 / Racial and cultural identification: not reported / current smokers	20 one-to-one interviews	Grounded theory
14	Glenn et al. (2017), Canada [60]	To consider how smoking among young adults relates to their local neighbourhood contexts to better understand place-based inequalities in smoking	*N* = 39 / Ages 18–25 / Gender identification: not reported / Racial and cultural identification: not reported / current smokers	9 focus groups	Thematic analysis
15	Grogan et al. (2009), England, UK [61]	To explore accounts of the impact of smoking on appearance in order to make suggestions for targeted appearance related anti-smoking campaigns aimed at young people	*N* = 87 / Ages 16–24 / Female: 48 / Male: 39 / Racial and cultural identification: not reported / current smokers and non-smokers	Focus groups	Thematic analysis and grounded theory
16	Haines et al. (2009), Canada [62]	To understand why young women smoke, using Bourdieu’s theory of cultural capital	*N* = 25 / Ages 16–18 / Female: 25 / White: 23 / Asian: 1 / Multiracial: 1 / current smokers	25 one-to-one interviews and participants asked to take photographs to discuss how smoking fits within their lives	Coding guided by a Bourdiesian theoretical framework
17	Hefler & Carter (2017), Australia [63]	To explore the intersection between stigmatised identity and smoking among young people who attended social services for at-risk youth in an inner city area of Australia	*N* = 18 / Ages 16–25 / Gender identification: not reported / Racial and cultural identification: not reported / current smokers, social smokers, ever-smokers, former smokers and never smokers	One-to-one interviews and follow-up interviews for respondent validation and to gather information about changes over time	Grounded theory
18	Hoek et al. (2011), New Zealand [64]	To explore how young adult social smokers view their smoking identities and the influences on social smoking	*N* = 13 / Ages 19–25 / Female: 4 / Male: 9 / New Zealand European: 10 / Maori: 2 / Australian: 1 / social smokers	13 one-to-one interviews	Thematic analysis
19	Hsia & Spruijt-Metz (2003), USA [65]	To identify what smoking means to Chinese American and Taiwanese American college students, and how those meanings may influence smoking behaviours in the context of acculturation	*N* = 30 / Ages 18–26 / Female: 16 / Male: 14 / Taiwanese American: 15 / Chinese American: 15 / current smokers, ever-smokers and never smokers	6 focus groups	Content analysis followed by data reduction and clustering
20	Jones et al. (2013), USA [66]	To identify factors influencing decisions to start smoking among young black African Americans in a US midwestern state	*N* = 22 / Ages 19–25 / Female: 16 / Male: 6 / Non-hispanic black: 22 / smokers, former smokers and non smokers	One-to-one interviews and follow-up interviews to verify the accuracy of data analysis	Thematic analysis
21	Kulbock et al. (2008), USA [67]	To discover potentially modifiable protective attitudes, beliefs and norms associated with decisions of non-smoking adolescents in the US	*N* = 39 / Ages 16–17 / Female: 22 / Male: 17 / African Americans: 22 / Caucasian Americans: 17 / non-smokers	Group interviews	Content analysis
22	McCool et al. (2013), New Zealand [68]	To understand how young adults perceive smokers and smoking in an environment non-accepting of smoking, and examine how this environment stigmatises smokers. The focus was on identity and stigma within a context of denormalising smoking policies in New Zealand	*N* = 86 / Ages 18–24 / Female: 49 / Male: 27 / New Zealand European: 43% / Maori: 37% / Pasifika: 28% / Asian: 5% / Other racial identity: 7% / smokers and non-smokers	14 group discussions and 4 one-to-one interviews	Thematic analysis
23	McQuoid et al. (2019), USA [69]	To explore the everyday smoking contexts and practices of bisexual young adults to reveal unique mechanisms driving tobacco use	*N* = 17 / Ages 18–26 / Cisgender female: 12 / Cisgender male: 2 / Gender queer (born female): 3 / White, Non-Hispanic: 7 / Hispanic/Latino: 6 / Asian American: 3 / African American: 1 / Native American: 1 / Southeast Asian/Pacific Islander: 1 / current smokers	Baseline survey, 30-day geographically explicit ecological momentary assessment (GEMA) integrated with follow-on one-to-one interviews	Thematic analysis
24	Nichter et al. (2006), USA [70]	To explore the gender dimensions of smoking among college students; specifically: acceptability of smoking, the monitoring of self and friends and norms of sharing and communicating	*N* = 35 / Ages 18–19 / Female: 17 / Male: 18 / Caucasian: 35 / current smokers: social smokers and casual smokers	2 ethnographic studies, including interviews, focus groups and observations of smoking on campus	Inductive and deductive analysis
25	Rosa & Aloise-Young (2015), USA [71]	To explore the smoker identity among US college student smokers, beyond the smoker/non-smoker dichotomy	*N* = 41 / Ages 18–21 / Female: 27 / Male or not specified: 14 / White: 80.5% / Hispanic: 9.7% / Other: 9.7% / daily smokers, social smokers and occasional smokers	6 focus groups	Thematic analysis
26	Scheffels (2009), Norway [45]	To explore young smokers’ construction of identity through their smoking experience in Norway	*N* = 21 / Ages 18–23 / Female: 10 / Male: 11 / Racial and cultural identification: not specified / current smokers	One-to-one interviews	Grounded theory and discourse analysis
27	Scheffels & Schou (2007), Norway [46]	To explore how young adult smokers in Norway talk about continuing to smoke in an increasing negative climate towards smoking, how they construct their identities, and the meaning they attribute to smoking as a choice	*N* = 21 / Ages 18–23 / Female: 10 / Male: 11 / Racial and cultural identification: not specified / current smokers	One-to-one interviews	Thematic and discourse analysis
28	Seguire & Chalmers (2000), Canada [72]	To explore the smoking patterns of late adolescent female smokers in Canada and factors which may/not be helpful in assisting them with quitting	*N* = 25 / Ages 17–19 / Female: 25 / Racial and cultural identification: not specified / current smokers and former smokers	One-to-one interviews	Codes and categories developed with reference to grounded theory
29	Wiltshire et al. (2005), Scotland, UK [48]	To explore experiences and attitudes towards smoking among mid to late adolescents, the role of smoking in their lives and the impact of their life transitions on their smoking	*N* = 99 / Ages 16–19 / Female: 52 / Male: 47 / Racial and cultural identification: not reported / daily smokers and social smokers	Mostly paired interviews, 4 three-person group interviews, 1 one-to-one interview	Thematic analysis
30	Youatt et al. (2015), USA [73]	To understand and explore specific factors influencing smoking among the young adult LBGTQ community	*N* = 30 / Ages 18–24 / Female: 30 / White: 24 / African American/Black: 4 / White/Latino/Other: 2 / Lesbian: 15 / Bisexual: 13 / Queer/Pansexual: 2 / current smokers, former smokers and never smokers	Telephone interviews	Thematic analysis

The conceptual categories and meta-themes generated from the data are listed in Table 2.

**Table 2:** Meta-themes within conceptual categories

Conceptual category	Meta-theme
1. Reasons for taking up smoking	1.1 Alleviating stress 1.2 Influence of family and friends 1.3 Transition to further study, employment or leaving home 1.4 Experimenting with other risk-taking behaviours
2. Adopting a smoker identity	2.1 Transforming identity: a mature image 2.2 Transforming identity: a rebellious image 2.3 Sense of belonging 2.4 Smoking in groups to prevent negative reactions 2.5 Smoking to facilitate new relationships 2.6 Alcohol and social smoking
3. Stigma of smoking	3.1 Sensing social disapproval 3.2 Women stigmatised for smoking
4. Barriers to quitting	4.1 Pleasure of smoking 4.2 Minimising the health risk of smoking 4.3 Not identifying with addiction 4.4 Not identifying as a smoker
5. Factors facilitating quit attempts	5.1 Health concerns 5.2 Self-motivation and self-confidence 5.3 Pregnancy 5.4 Recognising own addiction to smoking 5.5 Non-smoking peers

### Reasons for taking up smoking

#### Alleviating stress

 One of the main reasons young adults gave for taking up regular smoking was that smoking provided a coping mechanism for alleviating stress, frustration and boredom. Many felt it helped them cope with balancing responsibilities and problems such as financial hardship or unemployment [6, 48–50, 54, 56, 60, 63, 65, 68, 69, 71–73].

“We’re in college, that’s kind of stressful, you know we have jobs, we have social lives and we have school to worry about.” [73]

Three studies found that LGBTQ (Lesbian, Gay, Bisexual, Transgender, Queer/Questioning) young adults may start smoking due to factors which relate to their sexuality-related stressors, such as the stress of struggling with one’s sexual identity, sexual discrimination, prejudice or bullying [51, 69, 73]. Smoking was perceived as a tool for survival:

“Being queer in a heterosexist society is very stressful […] a lot of substance abuse within the queer community is directly tied to that stress” [51]

#### Influence of family and friends

The influence of family and friends who smoke was a key contributing factor for normalisation of smoking, leading individuals to choose to take up smoking [49, 53, 57, 58, 60, 63, 65–67, 72].

“My whole family smoked, I was brought up with it. I had to almost.” [72]

Studies of Black young adults and people of colour found culturally specific ways in which young adults initiated smoking. Where racial and cultural demographics have been reported, Black people and people of colour were studied in 13 of the 30 included papers [50–52, 58, 62, 64–69, 71, 73]. For African Americans in one study, smoking cannabis was a gateway to smoking tobacco [66], whereas young adults of colour explained how smoking tobacco is prevalent, socially accepted and encouraged [58, 65]:

“My culture [Latin American] doesn’t acknowledge that [smoking] is bad for them. It’s like, I’m going to die anyway.” [58]

Smoking was also found to reinforce a sense of community among LGBTQ people where smoking is a culturally accepted norm, particularly when socialising in gay bars and to seek social acceptance [51, 69, 73]

#### Transition to further study, employment or leaving home

Leaving home or the transition to university or college, with fewer restrictions around smoking and a smoking culture, enabled young adults to take up smoking. Some participants cited no longer having to conceal their smoking from their parents [48, 49, 53, 55–57, 73]:

“At school you were always getting watched but at college you’re not […] you’ve got the freedom to smoke.” [48]

#### Experimenting with other risk-taking behaviours

Smoking coincided with experimenting with other risk-taking behaviours, including sexual activity, drugs and alcohol [48, 56, 63, 72]:

“I don’t know if smoking led to drugs, but I think that’s what happened.” [72]

As socialising with friends in pubs, bars or clubs became a frequent occurrence, what was occasional experimentation with smoking developed into regular and heavier smoking:

“It was really just a gradual kind of increase, it wasn’t any kind of event that said I’m going to smoke more today. I think going out and going to clubs and having a drink and everything does kind of make you smoke more.” [56]

### Adopting a smoker identity

#### Transforming identity: A mature image

Smoking was perceived as a way to effectively self-manage one’s image and sense of belonging and to assist with transforming a young, naïve and conformist identity to appear confident, relaxed, in control, mature and rebellious and acquire enhanced social status [6, 45, 46, 49, 51, 53–55, 59, 62, 65, 67, 70–72].

Smoking was frequently reported to appear glamorous and provide a symbol of maturity for those wanting to look sophisticated, successful or mysterious [6, 45, 66, 70, 72]

“I remember feeling like an adult when smoking […] we always wanted to look older than we were.” [45]

For both men and women, cigarettes were used as a “fashionable prop” [59] or “social tool” to assist with confidence in social interactions [6, 46, 48, 51, 69, 72]. Some women thought that smoking facilitated weight control (i.e. nicotine suppressing one’s appetite) [59, 72]. Women in particular saw the act of smoking as a means of acquiring a mature social status during their transition to adulthood [45, 46, 59, 72], but as they became older their addiction to smoking became frustrating and some expressed regret for having started due to financial and health burdens:

“I can’t quit, we start young and we are hooked. One day you wake up in the morning and it’s too late.” [72]

#### Transforming identity: A rebellious image

Smoking also served to establish a rebellious image for those wishing to differentiate from other peer groups and appear unafraid to take risks, particularly within educational settings and among marginalised subcultures such as LGBTQ (Lesbian, Gay, Bisexual, Trans, Queer) communities [45, 49, 51, 69, 70, 72, 73].

“It’s like you smoke a cigarette… you do something you’re not supposed to be doing, you know. It’s like bad for you, but it’s also like a simple little rebellion.” [70]

Men in particular felt that smoking gave them social status and enhanced a “tough guy” or “bad boy” image [45, 46, 70]:

“It was cool you know. We gained status, and the girls thought it was tough […] It was fashion. It was a good investment.” [46]

LGBTQ people also reported smoking as supporting a tough image, but their motivation stemmed from wishing to protect themselves against physical violence and harassment if they otherwise were perceived as defenceless [51]

#### Sense of belonging

Becoming a smoker provided a sense of belonging and acceptance among peers, particularly for those in fear of rejection and alienation from social groups or with low self-esteem [6, 46, 48, 49, 51, 53, 55, 57, 60, 64, 70, 72, 73]:

“Rejection or alienation is a huge fear for adolescent girls […] that’s why I started, ‘cause everybody around me was smoking in my group, and I think that’s why I continued.” [59].

Young adults joining the workforce felt peer pressure to smoke as part of their working culture [56, 60]. For example, when working on a building site smoking provided an opportunity to take a break with your colleagues and feel part of the team [46, 48, 60]. Other workplace settings, such as hospitals, hotels and care homes, normalised smoking as the only way of enabling employees to take a rest break [49, 56, 60]:

“At the hotel you weren’t allowed a break unless you were a smoker” [56]

#### Smoking in groups to prevent negative reactions

Some studies noted the phenomenon of smoking in groups to enhance sociability, support each other and protect against negative reactions from within the group or from outsiders, rather than to be seen as a lone smoker, which was self-stigmatising [48, 49, 51, 57, 60, 70, 71]. Those who would not ordinarily smoke would smoke with their friends to be part of the smoking in-group:

“When properly all your mates smoke and when we sit around at ours and play cards, they’re like, send them out for a fag. We can’t really stay alone with those cards.” [49]

#### 2.5 Smoking to facilitate new relationships

There was evidence from many studies to suggest that smoking helped build relationships with others, made starting conversations easier and was a way to express empathy particularly in new school, work or social situations when meeting new people, without requiring the presence of alcohol [48, 49, 51, 53–55, 57, 59, 65, 66, 69–73]:

“You can hang out with people, smoke, and not be drinking as a social smoker.” [71]

#### Alcohol and social smoking

Numerous studies found a strong relationship between young adults’ alcohol consumption and their likelihood and frequency of smoking in social contexts [6, 45, 46, 50, 53–58, 63, 65–68, 70–72]. They noted that their frequency of smoking depended on the social context [45, 48]:

“When I am working I smoke maybe five a day. And at weekends, if we go to a bar, I can smoke 20 in one night just like that. And if I go to a party I can smoke up to 40.” [46]

Alcohol and parties were a catalyst for social smoking [45–47, 53–55, 57, 63, 64, 68, 70–73], where they felt less responsible for their actions, less risk-averse, and inclined to smoke more than usual to appear relaxed and curb nicotine cravings:

“After I’ve had a drink I just don’t care.” [64]

### Stigma of smoking

#### Sensing social disapproval

Sensing disapproval from others and being stigmatised as a smoker generated negative prejudice and hostility [45, 47, 50, 53–55, 57–60, 63, 65, 67, 68, 70, 72]. Smoking when not drinking or socialising was deemed by many young adults to be socially unacceptable. They regarded smoking to be acceptable only when drinking, otherwise it was stigmatised as an addiction [47, 54, 57, 70]. Participants reported receiving social disapproval from non-smoking peers or family members [46, 53–55, 65, 68, 72] or employers [48]:

“I don’t smoke at work ‘cause I work in a posh, posh shop and don’t want clients thinking ‘oh there’s another wee girl smoking’” [48]

#### Women stigmatised for smoking

Women smokers reported feeling particularly stigmatised [45, 50, 54, 58, 65, 70]. For some, the stigma of smoking around non-smoking friends or family led to them deciding not to smoke, thereby gaining their approval [53, 54, 67]. Women were stigmatised for smoking by men and women, smokers and non-smokers [45, 50, 54, 55, 58, 63, 65, 70]. Across studies women smokers were seen as being unladylike, unattractive, “trashy” and out of control, whereas male smokers appeared cool and in control:

“Smoking women look like sluts. They are wild and undisciplined.” [65]

Black women and women of colour were viewed particularly harshly, which exacerbated their stress and made them feel dehumanised:

“I feel that women that smoke cigarettes are looked down upon… like you can’t control yourself, you have a problem… you’re not really a woman if you smoke cigarettes.” [50]

To counter such prejudice, some women who smoked considered it important to smoke only at parties or in a “clean and controlled way” to maintain perceived femininity and social status [45]

### Barriers to quitting

Barriers to quitting included enjoying the act of smoking, minimising the health risks associated with being a smoker, not identifying with addiction or not identifying with “being a smoker”, even for those smoking on a regular or social basis [46–50, 52, 54, 57, 58, 64, 68, 70, 71]. Many were concerned with upholding their image of being an occasional smoker, felt they were exempt from associated health risks, and had a poor understanding of the nature of addiction and how it relates to them:

“If what I have gone through hasn’t killed me so far… the cigarette is probably not going to kill me” [49]

#### Pleasure of smoking

As an observation from only a few studies, some participants described the pleasure of smoking [46, 49] and not wanting to experience nicotine withdrawal, including cravings, increased sensitivity and irritability [48, 49, 72]. The act of smoking was often performed in a ritualistic or meditative manner which aided relaxation [49, 51]:

“Often when I get stressed or annoyed I just go and sort of calm down with a smoke” [49]

#### Minimising the health risk of smoking

For participants of a few studies, being otherwise fit and healthy [45, 46] or only smoking occasionally [54] were viewed as compensating for the health risk of smoking. This perpetuated denial of smoking related harms and rationalised the continuation of smoking:

“I can still take care of my health even if I smoke, because I just exercise and bother to be active” [46]

Others were unconcerned about their present health risk from smoking, even when experiencing breathing difficulties and throat problems, because they felt they were still young enough not to have to worry about cancer and smoking was still part of their socialisation [46, 53]

“I have sort of imagined that I will quit. Maybe it will help me that my brother is quitting now. He’s in his 30s, and that’s OK because then the risk is not so high, kind of. But if you’re 40 the risk of getting ill is a bit higher. Like, not many 30 year olds get lung cancer.” [46]

Quitting smoking was seen as a task for the future, and some participants planned to quit at an age milestone [46, 49, 53, 60, 68, 70, 72]:

“I don’t want to smoke my whole life. I want to quit by the time I’m 22 or something” [60]

Participants who felt socially disadvantaged reported that smoking mitigated mental health issues and was more accessible as a coping mechanism for stress than other forms of stress-relief, such as talking therapy or massage [51]:

“Working class people, folks of colour and queers and, God forbid, if you are all three of those things you are going to be smoking. You are stressed out. There are not a lot of things that are accessible to you in terms of relief. Like […] Who can afford mental health care? Sometimes smoking a cigarette is the difference between cutting myself or not” [51]

#### Not identifying with addiction

When smoking was seen as a habit or part of a routine it was not necessarily acknowledged to be an addiction because the decisions of whether and when to smoke were within young adults’ control [47, 48, 50]. Some smokers denied being addicted or unhealthy due to experiencing a lack of cravings or symptoms [48, 71]:

“Because I’ve never had an urge [to smoke]” [71]

#### Not identifying as a smoker

Many individuals did not identify with “being a smoker” and avoided classifying themselves in the smoking category [48, 52, 54, 64]. They believed they were not smokers because they only smoked socially [48, 52], only “borrowed” cigarettes from others rather than buying their own [22, 35], did not consider themselves to use habitually or within a daily routine [48, 52, 54], felt they could quit whenever they wanted to [46, 52] and could “go without” [48, 54, 64].

“I see a social smoker as someone who only wants a cigarette when they are having a few drinks and don’t smoke by themselves or have cravings during the day. Could go without.” [64]

Therefore, those who were “not a smoker” did not need to quit [52], smoking cessation was not a priority [47] and they perceived no reason to stop [54]. Cessation services, nicotine replacement therapy or counselling interventions were viewed to be “uncool” and aimed at older or addicted smokers rather than for young people [47, 58] and public health campaigns were thought to reinforce stigma felt by smokers, thereby alienating them [45]

“The people I know that have quit know about patches and gum and stuff, but they don’t want to use them. It’s a pride issue.” [58]

### Factors facilitating quit attempts

#### Health concerns

Health concerns and known health risks, such as cancer and other diseases, reinforced young adults’ decision not to smoke [48, 54, 58, 59, 65–67, 70, 72]. However, some young adults considered that developing a serious illness would lead them to quitting:

“Probably I‘d completely stop if I had cancer or anything like that.” [70]

They cited the negative cosmetic effects of smoking on physical appearance, such as aging skin, bad breath, stained teeth, tooth loss and odour [49, 54, 58, 59, 68, 72]. Some studies noted young adults’ reasons for attempting to quit smoking, including feeling short of breath, coughing and being generally unhealthy and unfit [65, 72]:

“I am trying to quit smoking because I find that my health and physique are not as good as they were.” [65]

#### Self-motivation and self-confidence

For young adults who anticipated a time when they would want to quit smoking, self-motivation and willpower [46, 57, 72] or going “cold turkey” [54, 72] were seen as the most effective quitting strategies [47, 58, 72]. This perspective is consistent with the role of self-agency in young adults who decided not to take up smoking because they saw themselves as confident and having a positive self-image [67] or being respected as a healthy role model among their peers [63]:

“I want to be a role model […] Like I’m well known and everything and I don’t smoke” [63]

#### Pregnancy

The most cited reasons women gave for considering quitting smoking in the future were becoming pregnant, the risk to their unborn foetus and the risk of second-hand smoke for young children [46, 49, 72]:

“When I’m pregnant I will quit.” [72]

Women perceived that it was socially unacceptable and irresponsible to smoke in pregnancy or around children, and it was particularly unacceptable to be seen to be smoking around your own children or in cars carrying children [49]:

“I’ve never understood that when you’re in the last stage of pregnancy, why you have to smoke in a public place. Can’t you do it somewhere out of sight?!” [49]

#### Recognising own addiction to smoking

An eventual recognition of one’s own addiction to smoking led to consequent quit attempts, although successfully quitting was more difficult than previously anticipated and some reported that their awareness of the problem came “too late” [48, 72]. Their former denial about being a smoker precluded their initial quit attempts:

“I never used to see myself as a smoker, it was just as someone who smoked. One day I couldn’t be bothered smoking and I wanted to stop but I couldn’t stop. And I didn’t even know I was addicted.” [48]

#### Non-smoking peers

Two studies found that some young adults transitioning to college, university or employment and making friends with non-smoking peers led to reducing their smoking or attempting to quit completely [56, 57]. Non-smoking friends, family and partners provided support for quitting smoking and e-cigarettes were successful used by some as a smoking cessation tool:

“When I bought the e-cigarette thing I bought him one for his birthday at the same time and he quit too” [57]

### Line of argument synthesis

**Fig. 2:**
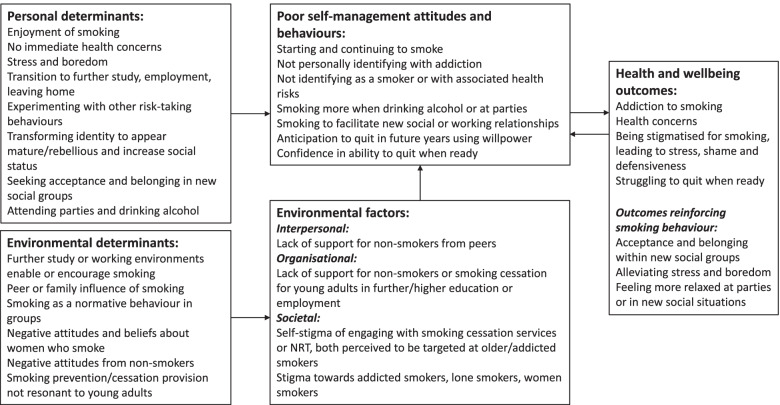
PRECEDE Logic model for young adults who smoke

Figure 2 depicts an overarching model for young adults starting to smoke and is based on the PRECEDE approach which defines the problem, its pathways and the ecological levels and influences on the problem [39, 74, 75]. This logic model serves as a conceptual framework presenting study participants’ smoking trajectories, including identity and lifestyle factors present within that phase of their lifecourse which (pre-)determines their smoking behaviour (such as identity transformation to increase social status) and the specific environmental determinants which influence and encourage smoking behaviour (such as smoking peers or family members) or reinforce stigma (negative attitudes towards women who smoke). The concurrent contexts of socialising at parties, with or without alcohol, or with new colleagues are synergistic with the environmental determinants of further study or working environments in which smoking is accepted as a normative activity by peers. The additional presence of alcohol in social contexts predisposes individuals who may not otherwise smoke to smoking. Individuals are likely to smoke more than usual when drinking alcohol due to lower inhibition control and to appear more relaxed and socially engaged within new social groups. Smoking during work breaks is normalised in various different occupational contexts, for many providing their only permitted break.

Family members, friends and colleagues who smoke may influence young adults’ decisions to start smoking, and conversely individuals from non-smoking families are at risk of smoking during their transition to living independently in the absence of being watched and experiencing familial disapproval. Many young adults begin smoking to alleviate stress. For LGBTQ people, Black women and women of colour in particular, being stigmatised led to smoking as a coping mechanism to deal with stress. For those with already stigmatised identities, smoking was performed as an act of resistance.

Unrealistic self-management attitudes and behaviours identified from the data reveal how smoking is initiated and maintained via young adults’ not identifying with addiction and anticipating that they will be able to quit in the future using willpower alone. They also smoke more when drinking alcohol and in social situations to facilitate new interpersonal relationships.

A number of environmental factors supported these attitudes and behaviours, ranging from the interpersonal to organisational and societal factors. A lack of support for non-smokers or smoking cessation interventions specifically tailored for young adults in further or higher educational settings or in the workplace is problematic, and presents a gap in public health service provision to be addressed. At the societal level, the data generated the meta-theme of *stigma* surrounding the engagement with smoking cessation services which were perceived to be targeted at older, “addicted” smokers and stigma towards those who were seen as addicted smokers, lone smokers and women smokers.

Regarding the perceived health and wellbeing outcomes associated with participants’ smoking behaviour, there were both negative outcomes (such as addiction to smoking and health concerns) and outcomes which reinforced smoking behaviour (such as acceptance and belonging in new social groups and alleviating stress and boredom). Not having any immediate health concerns associated with starting to smoke leads to a continuation of smoking and the minimisation or denial of the health risks associated with smoking. Furthermore, young adults’ anticipation of when they would quit smoking is at a milestone when they are older. The few who had made quit attempts reported struggling to quit on their own, and only then were they able to acknowledge their smoking habit as an addiction. Some successfully use e-cigarettes to quit smoking; however, the social benefits of smoking outweigh any immediate health side effects for the majority of young adults.

Young adults’ decisions to start and continue to smoke to develop desired social identities demonstrate that smoking is “socially addictive” and not just biologically addictive [6, 66]. This finding is congruent with Social Identity Theory which postulates that social identification with an ‘in-group’ is of emotional significance to an individual and that such membership enhances their self-esteem [76, 77]. Here, the process of in-group assimilation leads to pressure to conform to the in-group’s norms, i.e. peer pressure to smoke. In these instances, being a smoker becomes a positive identity and one that is socially reinforced, particularly for marginalised groups such as the LGBTQ community.

## Discussion

### Summary

This study is the first to review young adults’ experiences of starting and continuing to smoke as they construct their social identities whilst navigating the unstable transitions in their lifecourse towards adulthood. The ways in which young adults described their tobacco use were mediated by their experiences of the physical act of smoking, motivation for status enhancement within an in-group, and environmental contexts, including parties and workplaces where smoking was a normative behaviour. Many described smoking more frequently when socialising and when alcohol was present. Reasons stated for taking up smoking and becoming a smoker included alleviating stress, transforming one’s identity to appear mature or rebellious, and coping with the transition to further education, employment or leaving home. Gender differences were apparent, as women, particularly Black women and women of colour, were stigmatised for smoking by all genders (labelled as uncouth, promiscuous and unfeminine), and women considered pregnancy to be a key milestone for future cessation. Barriers to quitting smoking included young adults’ minimisation or denial of the health risks of their smoking, their enjoyment of smoking, and not identifying with addiction or with “being a smoker”. Smoking-related health problems were anticipated to be a future concern and many young adults felt that they would be able to stop smoking using self-determination alone when they were older. For the few who had attempted to quit smoking, the realisation of their addiction had become apparent to them “too late” and they struggled with quitting on their own. E-cigarettes were used as a cessation aid; however, smoking cessation services were disregarded as they were considered to be self-stigmatising and aimed at older or “addicted” smokers.

### Implications for policy makers, clinicians and future research

Consistent with previous research, smoker identities were found to be context-dependent and developed in congruence with young adults’ desire to appear mature and maintain social status [19].

Individuals’ abilities to self-regulate their behaviour and resist the temptation to smoke is inhibited by the presence of alcohol and when pleasure is derived from the act of smoking. Furthermore, even though future health problems from smoking, including the risk of cancer, are accepted among young adults, the immediate risk of smoking to one’s health was negated. Health concerns were rationalised as being a reason to quit in the future, not in the present, consistent with Cognitive Dissonance Theory’s dissonance resolution method of changing one’s beliefs about smoking-related harm so that continuing to smoke is no longer incongruent with those beliefs [78]. Psychosocial interventions could involve peer support around resisting the temptation to smoke in groups, particularly in new social settings, such as universities, colleges and the workplace. If the decision not to smoke is met with peer approval, then young adults are more likely to refrain from smoking and becoming regular smokers.

Considering young adults’ tenets that they may not be addicted to smoking and that smoking cessation services and nicotine patches and gum are shameful, “uncool” and aimed at older smokers, researchers and public health organisations should co-design new interventions with young adults that are specifically for them based on their perceived needs. Such interventions should resonate with their desire to quit in the future at key milestones, and address the social aspect of addiction, without reinforcing stigma, particularly for women and those of marginalised backgrounds [47, 58]. Culturally-sensitive health promotion interventions could be designed for those from diverse and disadvantaged backgrounds to minimise further health inequalities, and interventions may be specifically designed with and for Black people, women and LGBTQ communities [66, 73, 79]. Factors such as social isolation have recently been shown to play a larger role in smoking onset [80]. Furthermore, the development of future interventions should take into account smoking behaviours associated with the COVID-19 pandemic, during which smoking rates among young adults between 18 and 34 years have increased by 25% [81, 82]. Furthermore, a study examining the prevalence of psychological distress among smokers following the onset of the pandemic compared with previous years shows increased distress among smokers, particularly for women and those from more disadvantaged backgrounds, which may act as a barrier to cessation efforts and exacerbate health inequalities [83].

Rather than positioning interventions either within smoking prevention or smoking cessation frameworks, adopting a *harm reduction* approach for young adults may be a more appropriate strategy considering that they do not necessarily identify with or express themselves within the simple binary terms of “smoker” or “non-smoker” and addiction is not something to which many relate. For young adults, context is key and their smoking practices often fall into the fluid spaces between categories and definitions. Harm reduction interventions designed to speak to their emerging identities and lifestyles, whilst acknowledging the variance of perspectives and experiences of those with marginalised identities, may provide messages to young adults that reach them in meaningful and impactful ways. Future intervention approaches which may better resonate with young adults may include social marketing and education on tobacco industry tactics targeted at adolescents and the practice of refusing tobacco products in safe environments [84]. Helping young adults to recognise their addiction, and that they may not be able to quit as readily as they anticipate they will be able to, may be another effective harm reduction strategy and could facilitate earlier quit attempts. The role of e-cigarettes as an effective harm reduction tool providing nicotine replacement therapy may also be considered as an alternative to smoking among young adults [32, 85–87]. Proposed legislation changes in New Zealand for regulating vaping products aim to strike a balance between preventing the uptake of vaping among children and young adults, whilst supporting people who smoke to switch to a less harmful product (and thereby contributing to New Zealand’s ‘Smokefree 2025’ goal) [13]. Similarly, the European Union Tobacco Products Directive (TPD) has also introduced regulatory controls on e-cigarettes as well as setting out requirements on tobacco products, and the UK has enabled tobacco and e-cigarette regulations to continue to function following its withdrawal from the European Union [88].

With reference to the position some countries represented in this study have taken with regard to their ambitious tobacco control strategies, the prevention of smoking uptake among young adults in countries such as New Zealand (by way of phasing out tobacco entirely, so that people born after a certain date will never be able to legally purchase tobacco) will inevitably impact on young adults’ tobacco use, identities and pathways of smoking [13]. This contrasts with other countries (such as the UK and US) where tobacco control policy measures are taking less bold action [9, 11, 89].

### Strengths and limitations

A systematic and rigorous approach to conducting the meta-ethnography was employed, consistent with Noblit and Hare’s methodology and the eMERGe guidelines [16, 18], and we produced a new line of argument for young adults starting and continuing to smoke.

Most papers were rated as being of high or medium quality according to the CASP checklist [26]. Our strategy and process for data extraction and translating interpretations are described, including information on the processes conducted independently by reviewers and when data were checked for credibility and trustworthiness [18]. A limitation within the meta-ethnography is that most included studies were from the US, the UK and Australia; it would have been valuable to have had more representation from other Western European countries. A limitation associated with the primary studies included in the meta-ethnography is that the majority did not report racial and cultural identification of participants.

Primary studies from 2000 to 2020 were included in the synthesis, which is relevant to contemporary health policies, including the introduction of plain packaging and the smoking ban in enclosed public spaces [90, 91]. However, as health policy and practice vary between countries, participants’ experiences and accounts may be influenced by different contexts at policy-level, for example increased stigma towards smokers may be more salient in studies from countries where a smoking ban has been introduced.

There may be shifts that have occurred since this work was conducted as a result of the COVID-19 pandemic. Research suggests that during the pandemic smoking rates for young people in England increased along with smoking cessation activity, where a greater number of younger smokers made quit attempts during lockdown and more smokers quit successfully [82]. These findings coincide with the first ‘lockdown’ (legislation to restrict social interaction, including advice to stay at home), where a reduction in socialising may have led to less stimulus to continue smoking occasionally or socially without the predisposing contexts of social events or the presence of alcohol. The absence of these situations may have increased quitting behaviour and also the likelihood of success [82]. Another study found that people’s ability to stop smoking and their motivation to do so have been affected by new life stressors induced by the pandemic, and that those attempting to quit smoking reported lacking access to coping strategies previously available (such as visiting family and friends) [92].

## Conclusions

This meta-ethnography describing young adults’ pathways into smoking may provide a blueprint to inform the development of health and wellbeing interventions designed specifically for this target population. Future research may consider the evolving views of young adults within the 16–25 age bracket and beyond as they transition into adulthood, to include their changing perceptions of addiction and what it means to be a smoker as they become older and develop more concern for their health. Harm reduction approaches may resonate with young adults more than the smoking prevention / cessation binary conceptualisation.

## Supplementary Information

**Additional file 1.** PRISMA Flowchart of study selection.

**Additional file 2.** Medline Search Strategy.

**Additional file 3.** PRECEDE logic model for young adults who smoke.

## Data Availability

The protocol for the meta-ethnography was published in PROSPERO [93]. The search strategy can be accessed in Additional File 2. CASP quality checklists and ratings may be provided upon request to the corresponding author.

## Abbreviations

CASP: Critical Appraisal Skills Programme
LGBTQ: Lesbian, Gay, Bisexual, Trans, Queer/Questioning
