# Supplementary material for: Tobacco use, smoking identities and pathways into and out of smoking among young adults: a meta-ethnography
Source: Subst Abuse Treat Prev Policy. 2022 Mar 28;17:24. doi: 10.1186/s13011-022-00451-9 (PMC8960094; doi:10.1186/s13011-022-00451-9)
Supplement: Supplementary file 1 — Additional file 1. PRISMA Flowchart of study selection. [file 13011_2022_451_MOESM1_ESM.docx]

**Additional File 1:**

**Figure 1. PRISMA flowchart of study selection**


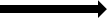


Records identified through database searching (n = 3230)

Duplicate records removed (n = 1234)

Papers excluded (n = 1879)

Papers excluded with reasons (n = 87)

- 14 had insufficient 1^st^ order data
- 73 did not meet the inclusion criteria

Full texts screened for eligibility (n = 117)

Titles and abstracts screened (n = 1996)

Flow chart

Papers included in meta-ethnography (n = 30)
