# Supplementary material for: Tobacco use, smoking identities and pathways into and out of smoking among young adults: a meta-ethnography
Source: Subst Abuse Treat Prev Policy. 2022 Mar 28;17:24. doi: 10.1186/s13011-022-00451-9 (PMC8960094; doi:10.1186/s13011-022-00451-9)
Supplement: Supplementary file 2 — Additional file 2. Medline Search Strategy. [file 13011_2022_451_MOESM2_ESM.docx]

**Additional File 2: Medline Search Strategy**

| **#** | **Searches** | **Results** |
| --- | --- | --- |
| 1 | (teen or teens or teenage*).tw. | 23946 |
| 2 | adolescen*.tw. | 199905 |
| 3 | (youth or youths).tw. | 47651 |
| 4 | ((young adj (person* or people or adult*)) or early adult*).tw. | 91043 |
| 5 | (student or students).tw. | 198273 |
| 6 | adolescent/ | 1851152 |
| 7 | or/1-6 | 2072789 |
| 8 | "Tobacco use"/ | 811 |
| 9 | *Smoking/ | 73289 |
| 10 | "Tobacco Use Disorder"/px [Psychology] | 2640 |
| 11 | Tobacco products/ | 2152 |
| 12 | Vaping/ | 39 |
| 13 | (smoke or smoking or smoker or smokers).tw. | 211434 |
| 14 | (vape or vaping or vaper or vapers).tw. | 159 |
| 15 | (cigarette* or cigar or cigars or cigarillo*).tw. | 56939 |
| 16 | ("tobacco use" or "nicotine use").tw. | 12226 |
| 17 | or/8-16 | 231279 |
| 18 | Qualitative research/ or Phenomenology/ or Focus groups/ | 52080 |
| 19 | (qualitative or ethnograph* or grounded theory).tw. | 152937 |
| 20 | (("semi-structured" or semistructured or unstructured or informal or "in-depth" or indepth or "face-to-face" or structured or guide) and (interview* or discussion*)).tw. | 87378 |
| 21 | focus group*.tw. | 28344 |
| 22 | interviews as topic/ | 53021 |
| 23 | ((audio-recorded or transcribed) and (interview* or survey* or questionnaire*)).tw. | 8543 |
| 24 | (themes adj3 (identif* or explor*)).tw. | 8772 |
| 25 | or/18-24 | 266415 |
| 26 | (identit* or reflecti* or meaning* or attitude* or perception* or perceiving*).tw. | 584308 |
| 27 | (self adj (concept or identit* or identif* or labe*)).tw. | 7955 |
| 28 | Attitude/ | 44341 |
| 29 | Self concept/ | 52545 |
| 30 | Social perception/ | 20535 |
| 31 | social identification/ | 8018 |
| 32 | or/26-31 | 661280 |
| 33 | 7 and 17 and 25 and 32 | 609 |
| 34 | limit 33 to (english language and yr="1998 -Current") | 555 |
