# Supplementary material for: Tobacco use, smoking identities and pathways into and out of smoking among young adults: a meta-ethnography
Source: Subst Abuse Treat Prev Policy. 2022 Mar 28;17:24. doi: 10.1186/s13011-022-00451-9 (PMC8960094; doi:10.1186/s13011-022-00451-9)
Supplement: Supplementary file 3 — Additional file 3. PRECEDE logic model for young adults who smoke. [file 13011_2022_451_MOESM3_ESM.pptx]

## Slide 1
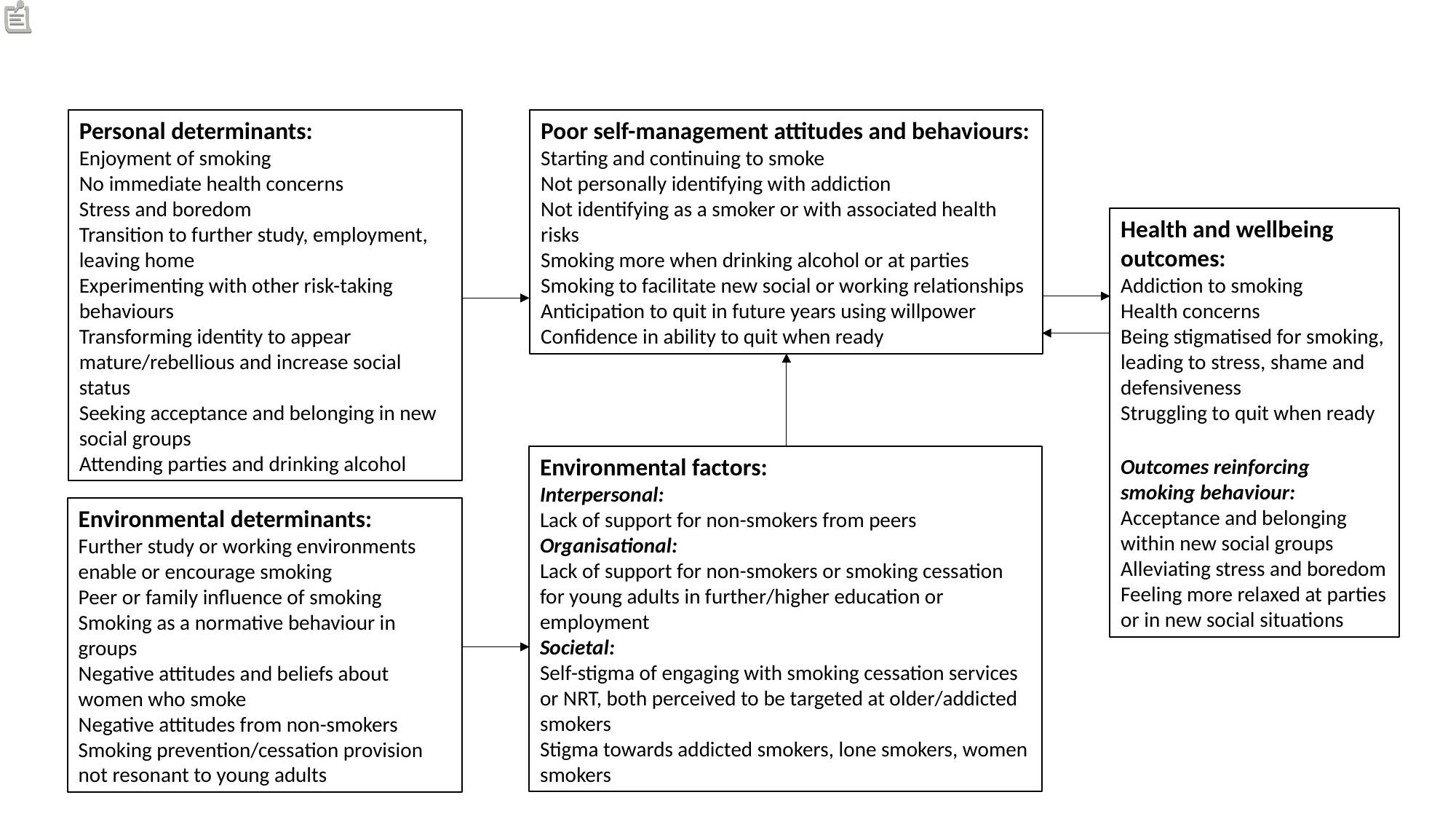

Personal determinants:
Enjoyment of smoking
No immediate health concerns
Stress and boredom
Transition to further study, employment, leaving home
Experimenting with other risk-taking behaviours
Transforming identity to appear mature/rebellious and increase social status
Seeking acceptance and belonging in new social groups
Attending parties and drinking alcohol
Poor self-management attitudes and behaviours:
Starting and continuing to smoke
Not personally identifying with addiction
Not identifying as a smoker or with associated health risks
Smoking more when drinking alcohol or at parties
Smoking to facilitate new social or working relationships
Anticipation to quit in future years using willpower
Confidence in ability to quit when ready
Health and wellbeing outcomes:
Addiction to smoking
Health concerns
Being stigmatised for smoking, leading to stress, shame and defensiveness
Struggling to quit when ready
Outcomes reinforcing smoking behaviour:
Acceptance and belonging within new social groups
Alleviating stress and boredom
Feeling more relaxed at parties or in new social situations
Environmental factors:
Interpersonal:
Lack of support for non-smokers from peers
Organisational:
Lack of support for non-smokers or smoking cessation for young adults in further/higher education or employment
Societal:
Self-stigma of engaging with smoking cessation services or NRT, both perceived to be targeted at older/addicted smokers
Stigma towards addicted smokers, lone smokers, women smokers
Environmental determinants:
Further study or working environments enable or encourage smoking
Peer or family influence of smoking
Smoking as a normative behaviour in groups
Negative attitudes and beliefs about women who smoke
Negative attitudes from non-smokers
Smoking prevention/cessation provision not resonant to young adults
